# Demographic associations with plasma biomarkers in the oldest‐old

**DOI:** 10.1002/alz70861_109011

**Published:** 2025-12-23

**Authors:** Christina B. Young, Hilary L. Colbeth, Paola Gilsanz, María M. M. Corrada, Batool M. Rizvi, Sitong Zhou, Charles De Carli, Lee‐Way Jin, Rachel A. Whitmer

**Affiliations:** ^1^ University of California, Davis, Sacramento, CA USA; ^2^ University of California, Davis, Davis, CA USA; ^3^ Kaiser Permanente Northern California Division of Research, Pleasanton, CA USA; ^4^ University of California, Irvine, Irvine, CA USA; ^5^ University of California Davis Medical Center, Sacramento, CA USA; ^6^ University of California, Davis School of Medicine, Sacramento, CA USA

## Abstract

**Background:**

Although plasma biomarkers are becoming increasingly common for the diagnosis of Alzheimer’s disease, an understanding of these biomarkers in diverse cohorts particularly in the oldest‐old is unclear. This study examines the association of plasma biomarkers with age, sex, race/ethnicity, and APOE genotype in the LifeAfter90 cohort, a population‐based sample of those over 90 years old.

**Method:**

Plasma biomarkers from 226 non‐demented participants in the Life After 90 Study were measured on the Quanterix Simoa platform. Participants had a mean (SD) age of 91 (SD: 1.6) years, were 63% (*n* =142) female, and were from diverse race/ethnic backgrounds [27% White (*n* =61), 20% Black (*n* =42), 20% LatinX (*n* =47), 28% Asian (*n* =64), 4% Other/Multiple (*n* =8)]. Blood samples were analyzed for AB42/AB40, pTau‐181, pTau‐217, GFAP, NfL, and APOE genotype. General linear models were used to examine age, sex, and race/ethnicity effects on each plasma biomarker; another set of models that additionally included the interaction of race/ethnicity and APOE‐e4 carrier status was used to examine race‐specific effects of APOE‐e4.

**Result:**

There were no significant age associations in this cohort of participants over 90 years old, likely because of the restricted age range. Females had significantly lower pTau181 levels than males, but there was no sex effect on pTau217 or AB42/AB40. There were no significant differences between racial/ethnic groups or no significant race/ethnicity by APOE‐e4 status interactions.

**Conclusion:**

Plasma biomarkers are largely unaffected by demographic variables in the oldest old.

